# Application of Zero-Valent Iron Nanoparticles for the Removal of Aqueous Zinc Ions under Various Experimental Conditions

**DOI:** 10.1371/journal.pone.0085686

**Published:** 2014-01-09

**Authors:** Wen Liang, Chaomeng Dai, Xuefei Zhou, Yalei Zhang

**Affiliations:** 1 State Key Laboratory of Pollution Control and Resources Reuse, Tongji University, Shanghai, China; 2 College of Civil Engineering, Tongji University, Shanghai, China; RMIT University, Australia

## Abstract

Application of zero-valent iron nanoparticles (nZVI) for Zn^2+^ removal and its mechanism were discussed. It demonstrated that the uptake of Zn^2+^ by nZVI was efficient. With the solids concentration of 1 g/L nZVI, more than 85% of Zn^2+^ could be removed within 2 h. The pH value and dissolved oxygen (DO) were the important factors of Zn^2+^ removal by nZVI. The DO enhanced the removal efficiency of Zn^2+^. Under the oxygen-contained condition, oxygen corrosion gave the nZVI surface a shell of iron (oxy)hydroxide, which could show high adsorption affinity. The removal efficiency of Zn^2+^ increased with the increasing of the pH. Acidic condition reduced the removal efficiency of Zn^2+^ by nZVI because the existing H^+^ inhibited the formation of iron (oxy)hydroxide. Adsorption and co-precipitation were the most likely mechanism of Zn^2+^ removal by nZVI. The FeOOH-shell could enhance the adsorption efficiency of nZVI. The removal efficiency and selectivity of nZVI particles for Zn^2+^ were higher than Cd^2+^. Furthermore, a continuous flow reactor for engineering application of nZVI was designed and exhibited high removal efficiency for Zn^2+^.

## Introduction

Zinc is one of the trace elements closely related to human health. It is essential for living organisms [1]. But excessive amount of zinc in the environment is toxic to man, animals and plants. When the concentration of zinc increases above a limit, it may lead to acute gastroenteritis, peritonitis, growth retardation and even shock or death [2]–[4]. Zinc toxicity to aquatic organisms and ecosystems has been frequently reported [5], [6]. Excessive zinc may lead to the death of fishes [7]. Irrigation water containing excessive zinc may cause poor crop growth and affect the health of the eaters [8], [9]. The presence of zinc is mainly from industrial pollution, such as galvanizing plants, pigments, mine drainage, etc. Zinc is commonly detected in the aquatic environment with its widely use in industry [10]. Considering its toxicity and non-biodegradability, it is necessary to effectively remove zinc. Current main zinc removal techniques from aqueous solutions include physico-chemical precipitation, ion exchange, complexation, adsorption, electrodialysis, etc. [11]–[13].

Nanoscale zero-valent iron (nZVI) has been investigated as a new tool for the reduction of contaminated water and soil for more than 10 years, and the technology has been applied in many countries worldwide. The nZVI has been proven as a highly effective technology for the removal or degradation of various chemical pollutants, such as β-lactam and nitroimidazole based antibiotics [14], azo dyes [15], chlorinated solvents [16], chlorinated pesticides [17], organophosphates [18], nitroamines [19], nitroaromatics [16], alkaline earth metals [20], transition metals [21], [22], post-transition metals [21], [22], metalloids [21], actinides [21], etc. The successful application of nZVI in dissolved metals removal was explored and reported by many researchers [23].

The determined contaminant removal pathways of nZVI include adsorption, complexation, (co)precipitation and surface-mediated chemical reduction [24]. The removal mechanism by nZVI mainly involves adsorption/surface complexation for metal ions such as Zn^2+^ and Cd^2+^ which have the standard electrode potentials (E^0^) for reduction to a metallic state that are very close to, or more negative than Fe^0^ (−0.44 V). For metal ions such as Hg^2+^ and Cu^2+^ whose E^0^ are much more positive than that of Fe^0^, removal of metal ions is mainly realized via surface-mediated reductive precipitation in comparison. While metal cations are only slightly more electropositive than Fe^0^, the removal is mainly realized via the adsorption with partial chemical reduction [22].

In this study, the removal mechanism of Zn^2+^ by nZVI was investigated based on the operation conditions, including nZVI solids loading, pH value and dissolved oxygen (DO). The X-ray Photoelectron Spectroscopy (XPS) of nZVI was performed to detect the valence of zinc and iron to determine whether chemical reaction happened. Furthermore, a continuous flow reactor was designed and applied to remove Zn^2+^ for evaluating the engineering application of nZVI.

## Materials and Methods

### Chemicals and Materials

Zinc chloride (ZnCl_2_), analytic grade cadmium acetate (Cd[CH_3_COO]_2_•2H_2_O), sodium borohydride (NaBH_4_,98%) and ferric chloride anhydrous (FeCl_3_) were purchased from Aladin (Shanghai, China). Hydrochloric acid (HCl), sodium hydroxide (NaOH), nitric acid (HNO_3_) and anhydrous ethanol (C_2_H_5_OH) were obtained from Sinopharm Chemical Reagent Shanghai Co., Ltd. (Shanghai, China). All chemicals were used without further purification.

Deionized water was prepared with a Milli-Q water purification system (Millipore, Bedford, MA, USA). Microporous membranes (0.22 µm×50 mm) were obtained from CNW (Germany).

### Synthesis of nZVI

The nZVI was synthesized according to the method of liquid-phase reduction of ferric trichloride by sodium borohydride [25]. The sodium borohydride (NaBH_4_, 0.5 M) and ferric chloride anhydrous (FeCl_3_, 0.1 M) with the volume ratio of 1∶1 were vigorously reacted. Then the generated jet-black nZVI particles were collected through vacuum filtration and respectively washed with deionized water and anhydrous ethanol for three times. Finally, fresh nZVI particles were stored in anhydrous ethanol solution at 4°C in order to avoid oxidization prior to use.

### Characterization of nZVI

Samples of nZVI were prepared by depositing a few droplets of ethanol-diluted nZVI solution onto a carbon-coated transmission electron microscopy (TEM) grid in an oxygen-limiting chamber. But the samples were exposed to air transitorily during transfer from the oxygen-limiting chamber to the microscope. The high-resolution TEM observation was performed using a JEOL JEM 2011 HR-TEM operated at 200 kV with an INCA EDS system.

The specific surface area of nZVI was measured by BET analysis.

The nZVI particles were dried in a refrigerated drying chamber and then kept under seal at 4°C for X-ray photoelectron spectroscopy (XPS) measurement and X-ray diffraction (XRD) measurement. The XPS spectra were obtained with a Perkin Elmer PHI 5000 ESCA System under Al Kα radiation at 1486.6 eV to study the conversion of the element contents and valence states on nZVI surface. The XRD was carried out on a Bruker X-ray D8 Advance diffraction instrument (Cu Kα) and the diffraction angle (2θ) from10 to 90° was scanned.

### Batch Experiments

A 100 mg/L stock solution of ZnCl_2_ was prepared with deionized water. Uptake reactions were initiated by the addition of nZVI particles into 100 mL aliquots of Zn stock solution. The nZVI loading concentration in the solution was 0.1, 0.2, 0.3, 0.4, 0.6, 0.8, 1.0, 1.2, 1.4, 1.6, 1.8 and 2.0 g/L, respectively, at a zinc ion concentration of 100 mg/L. After mixing, the reactors were continuously shaken for 2 hours on an orbital shaker. The optimum loading of nZVI was obtained by comparing the results of the above experiments. All the experiments were performed in triplicate.

The effect of oxygen on Zn^2+^ removal by nZVI was investigated under the oxygen-limiting and oxygen-contained conditions with the optimum nZVI loading. The oxygen-limiting condition was established by flowing nitrogen over the solution. The initial solution pH value was controlled at 5. Reaction time was 5, 10, 15, 20, 25, 30, 40, 50, 60, 70, 80, 90 and 100 min, respectively. All the experiments were performed in triplicate.

To investigate the effect of solution pH on the Zn^2+^ removal by nZVI, the initial solution pH was adjusted from 3 to 5 with the initial Zn^2+^ concentration at 100 mg/L by small amounts of HCl or NaOH solution. Then water samples with different pH values were applied to 1 g/L nZVI. All the experiments were performed in triplicate.

To investigate the effect of cadmium on Zn^2+^ removal by nZVI, three different water samples were used. Sample 1 contained 100 mg/L Zn^2+^ solution. Sample 2 contained 100 mg/L mixture of Zn^2+^ Cd^2+^. Sample 3 contained 100 mg/L Cd^2+^ solution. The uptake experiments were conducted with the optimum loading of nZVI for 2 h. All the experiments were performed in triplicate.

All solution samples were filtered with 0.22 µm membrane acidified with 4% ultrahigh purity HNO_3_ before analysis. Zinc and iron in the sample were determined by inductively coupled plasma optical emission spectrometry (ICP-OES, PerkinElmer Optima 2100 DV, USA).

### Experiment in Continuous Flow Reactor

**Figure 1:**
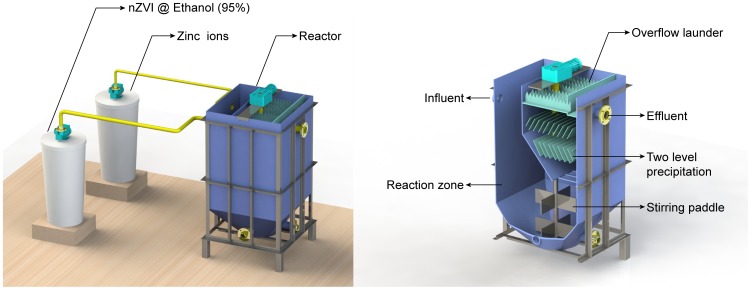
The continuous flow reactor. A continuous flow reactor was designed to realize the continuous removal of Zn^2+^ by nZVI.

A continuous flow reactor was designed to realize the continuous removal of Zn^2+^ by nZVI (Figure 1). The reactor was composed of reaction zone and precipitation zone, with the dimension of 0.2 m length, 0.2 m width, and 0.5 m height. The tank with inclined-plate could enhance the solid-liquid separation. The hydraulic retention time in the reaction zone was set to be 1 h and the nZVI solids concentration was set to be 1.0 g/L. The solution of Zn^2+^ with a concentration of 15 mg/L was stored in a reservoir tank and flowed to the reactor with a peristaltic pump at 120 mL/min. The design of two level precipitations made the nZVI particles settling to the reaction zone, so that the nZVI particles could be reused. The effluent was periodically sampled for analysis.

### Statistical Analyses

One-way ANOVA was performed to assess the removal efficiency of Zn^2+^ by nZVI. Statistical significance was evaluated at *p*<0.05 level. All statistical analyses were performed with SPSS software (Ver 13.0; SPSS, Chicago, IL, USA). The experimental data were expressed as mean±standard deviation (SD).

## Results and Discussion

### Characterization of nZVI

**Figure 2:**
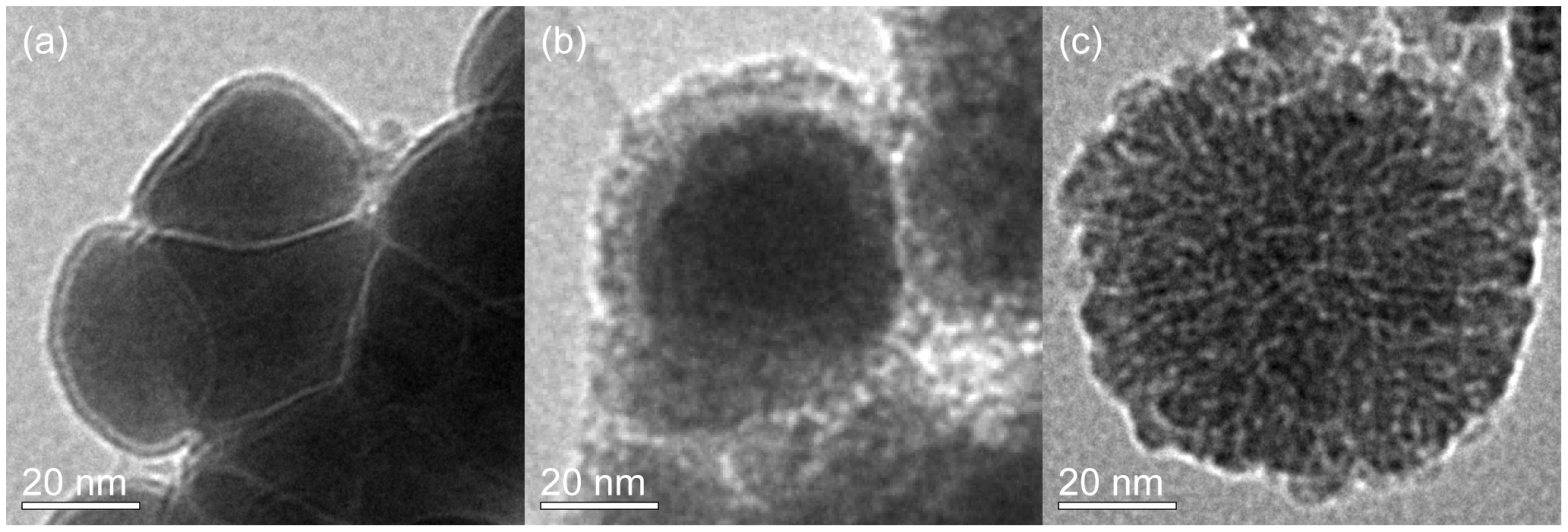
The TEM analysis of nZVI. Three kinds of nZVI particles were analyzed by TEM: (a) the fresh nZVI particles, (b) the stock nZVI after the reaction with Zn^2+^, and (c) the stock nZVI as a blank control sample.

Three kinds of nZVI particles were analyzed by transmission electron microscopy (TEM). The fresh nZVI particles were shown in Figure 2(a). The smooth sphere indicated that little oxidation happened on the surface. The nZVI particles were typically less than 100 nm in diameter. As shown in Figure 2(b), the stock nZVI after the reaction with the concentration of 100 mg/L Zn^2+^ in 2 h, appeared a single particle composed of a dense core surrounded by a thin amorphous shell, which indicated that the reaction occurred on the surface of nZVI, and that a core-shell structure was formed during the reaction. As shown in Figure 2(c), the stock nZVI as the blank control showed a complete oxidation. The core structure disappeared due to corrosion, which indicated that the reaction on the surface of nZVI could protect Fe-core from further corrosion.

The specific surface area of the nZVI sample was measured by BET analysis. The analysis results indicated that the specific surface area of the nZVI sample was 18.9887 m^2^/g, which was much higher than that of ZVI, 0.048 m^2^/g [26]. The high specific surface area of nZVI demonstrated its high adsorption capacity.

**Figure 3:**
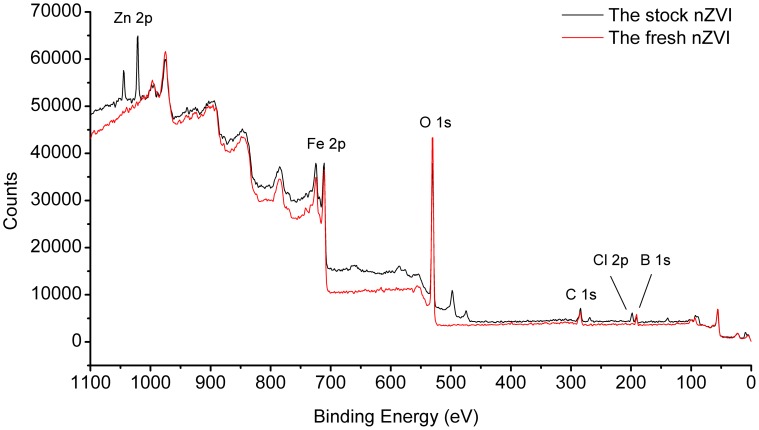
The XPS full scan analysis of nZVI.

The XPS spectra were also obtained to study the elements on the surface of nZVI. As shown in Figure 3, Zn and Cl were found on the surface of nZVI after the reaction.

### Effect of nZVI Solids Concentration

**Figure 4:**
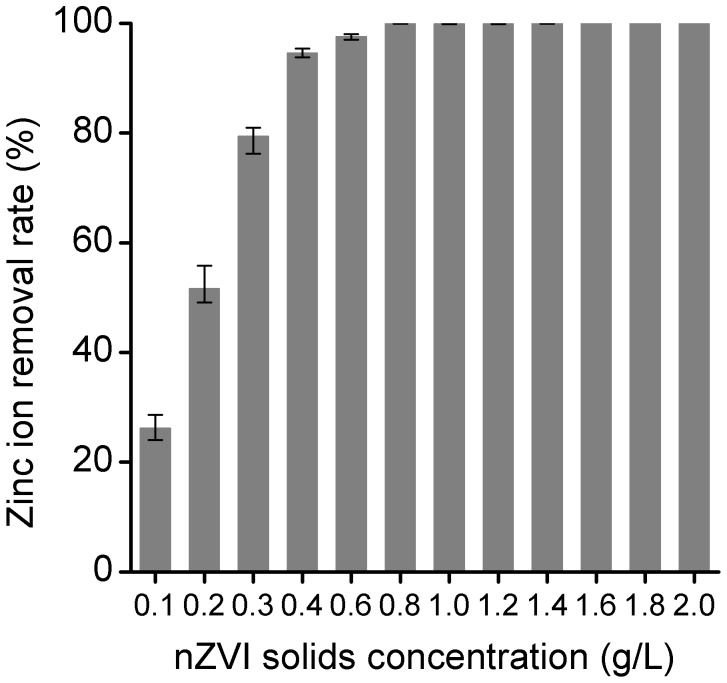
Effect of nZVI solids concentration on Zn^2+^ removal.

The uptake experiments were conducted with 0.1 to 2 g/L nZVI at a zinc ion concentration of 100 mg/L, respectively for 2 h. As shown in Figure 4, the removal efficiency of Zn^2+^ was increased with the increase of nZVI loading. The added Zn^2+^ was completely removed under the nZVI loading of 0.8 g/L or higher. When nZVI loading was higher than 1.0 g/L, the removal efficiency of Zn^2+^ remained about 99%. Higher loading of nZVI could provide more surface area, which enhanced the Zn^2+^ removal efficiency by nZVI. Thus, we proposed that 1 g/L nZVI was the optimum solids concentration of nZVI required for complete removal of 100 mg/L Zn^2+^ under the examined experimental conditions. And the concentration ratio was adopted in the subsequent experiments.

The high removal efficiency of Zn^2+^ by nZVI had been proved by Weile Yan [27]. The determined removal pathways of the contaminant mainly included adsorption, complexation, (co) precipitation and surface-mediated chemical reduction [24]. The standard reduction potential of zinc E^0^ (Zn^2+^/Zn) is −0.76 v, while the standard reduction potential of iron E^0^ (Fe^2+^/Fe) is −0.44 v. The ionization tendency of zinc is higher than iron. So Zn^2+^ removal by nZVI is likely not caused by the surface-mediated chemical reduction.

### Effect of Dissolved Oxygen

**Figure 5:**
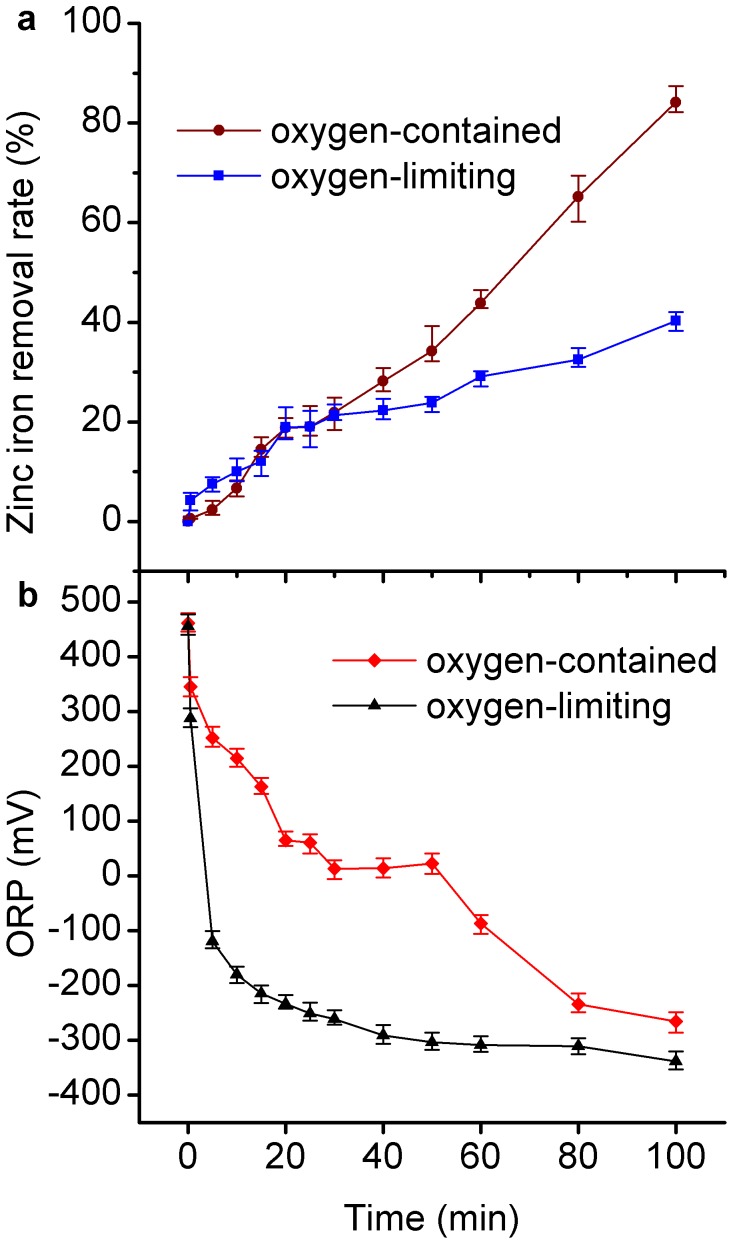
The Zn^2+^ removal and ORP in oxygen-contained and oxygen-limiting conditions.

The effect of dissolved oxygen (DO) on Zn^2+^ removal was examined to further study the removal mechanism on the surface of nZVI particles. The freshly prepared 1.0 g/L iron particles were injected into the solution with a zinc ion concentration of 100 mg/L in two 3-neck flasks. One of the flasks had an oxygen-limiting condition with the DO concentration below 0.5 mg/L, but the other one had an oxygen-contained condition with the DO concentration above 5.0 mg/L. As shown in Figures 5(a) and 5(b), the removal extent of Zn^2+^ under oxygen-limiting condition was up to 40%, but a higher removal extent of 80% appeared under oxygen-contained condition. Under both conditions, the Zn^2+^ removal extent increased to 25% in the first 30 min. After that, the removal trend became different. Under oxygen-limiting condition, another 15% of Zn^2+^ removal extent was achieved. Under oxygen-contained condition, the removal extent reached 80%. Meanwhile, the range of oxidation-reduction potential (ORP) under the two conditions presented different processes. Under the oxygen-limiting condition, the value of ORP dropped rapidly, and then came to a gentle decline in the residual contact time. In contrast, under the oxygen-contained condition, the value of ORP decreased in the first 30 min, remained unchanged for the next 20 min, and then decreased in the residual contact time. The change processes of ORP indicated that complex redox reactions occurred. The initial drop of ORP was likely caused by the consumption of dissolved oxygen during the oxidation of the Fe (0). The slower decline of ORP under oxygen-contained condition was caused by the supply of oxygen from atmosphere. The above results declared that DO was one of the important factors for the removal of Zn^2+^ by nZVI. As mentioned above, Zn^2+^ removal by nZVI is likely not caused by the surface-mediated chemical reduction. The result that nZVI particles were corroded and oxidized by DO might be interpreted as follows: the particles were covered by iron (oxy) hydroxide on the surface and the zinc ion was adsorbed on iron (oxy) hydroxide.

Under the oxygen-limiting condition, the predominant electron receptors should be water and the corrosion reaction could occur as follows [25], [26]:
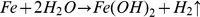
(1)

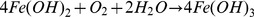
(2)


Under the oxygen-contained condition, the corrosion reaction could occur as follows [28]:
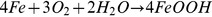
(3)


Heavy metal could be adsorbed by Fe(OH)_3_ and FeOOH. Nano/mico goethite has been proved to be efficient with variable capabilities in the removal of five metal ions including Zn^2+^ from aqueous solution [29]. Removal of arsenic from water with granular ferric hydroxide has been discussed [30], [31]. The reason for low Zn^2+^ removal extent under the oxygen-limiting condition could be found in Equation (2). Under oxygen-limiting condition, the formation of Fe(OH)_3_ was restricted by the oxygen supply, thus leading to the low removal efficiency.

The result that nZVI particles was corroded and oxidized into ferric ion by DO might be interpreted as follows: the particles were covered by iron hydroxide precipitation on their surface and the zinc ion was adsorbed on and/or co-precipitation on iron hydroxide.

### Effect of Solution pH

**Figure 6:**
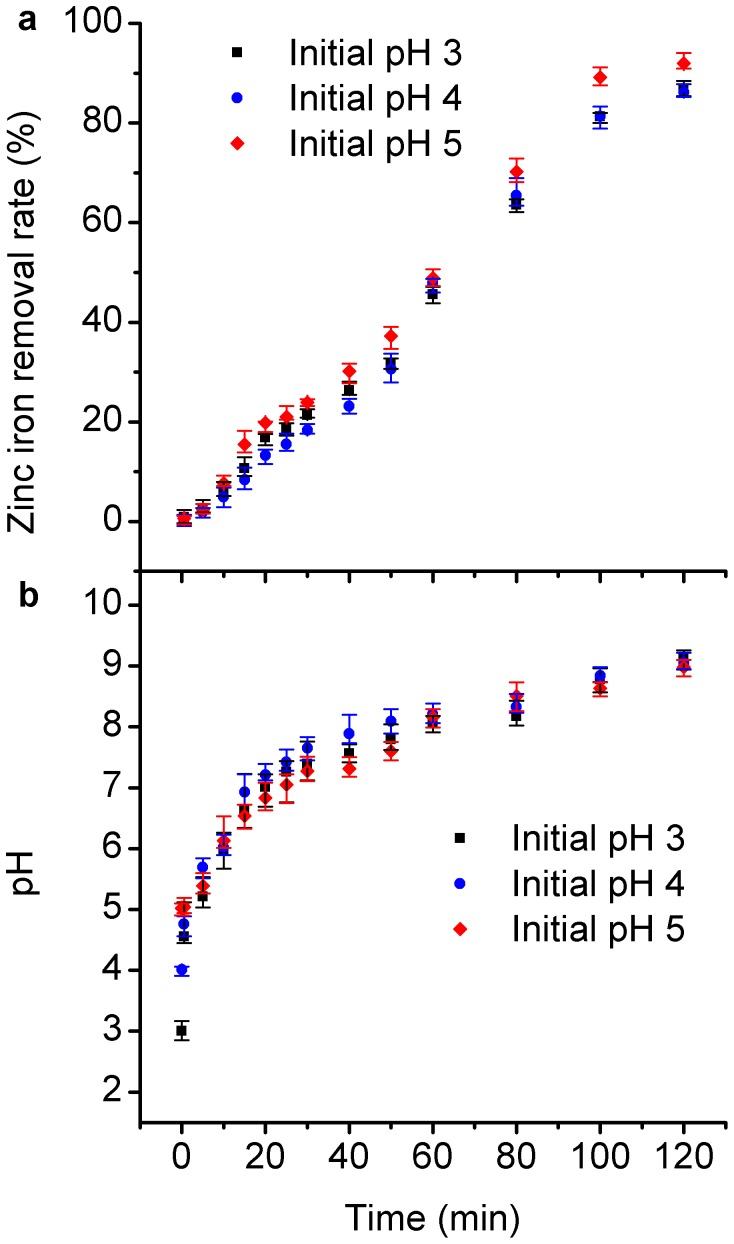
Effect of pH value on Zn^2+^ removal.

The pH is an important factor for Zn^2+^ removal by nZVI. The freshly prepared 1.0 g/L iron particles were injected into the solution with a zinc ion concentration of 100 mg/L within 2 h. Uptake results at various pH conditions were shown in Figure 6(a). Some floccose sediment generated when the pH value of the stock zinc ions solution was above 8. Thus, the pH values of 3, 4 and 5 were selected. The higher removal efficiency was observed in the experiment with an initial pH value of 5. The percentage of uptake of Zn^2+^ rose gradually with an increase in pH. The similar effect of pH value has been elucidated by Kishimoto [26]. The variation of pH under three initial values was shown in Figure 6(b). The pH value rose with the contact time. The final pH values were above 8.5 in spite of different initial values. In most part of the corrosion process, the pH value was above 7, while the cementation, which was caused by the glued sediment particles, process was highly effective under acidic conditions in the absence of DO [23]. Thus, the cementation role was not the main effect in Zn^2+^ removal.

With the change in pH value, iron was corroded by acid and oxygen. During the corrosion of iron by acid, Equations (4), (5) and (6) could happen [25], [26]. The H^+^ inhibited the formation of iron (oxy)hydroxide, resulting in the low removal extent of Zn^2+^ by nZVI.
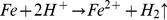
(4)

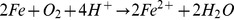
(5)

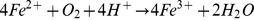
(6)


Equations (7), (8) and (9) could happen with the rise in pH value during oxygen corrosion [25], [26], [28]. Large iron (oxy)hydroxide could adsorb Zn^2+^ on the surface.
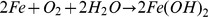
(7)

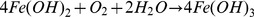
(8)

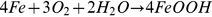
(9)


### Removal Mechanism

The heavy metal removal by nZVI generally involves redox, cementation, adsorption and precipitation. The standard reduction potential of zinc E^0^(Zn^2+^/Zn) is −0.76 v, while the standard reduction potential of iron E^0^(Fe^2+^/Fe) is −0.44 v. Therefore, it is not true that Zn^2+^ removal by nZVI is achieved due to the higher ionization tendency of zinc than that of iron. The cementation is usually effective under acidic pH without DO [23]. In this study, removal efficiency of Zn^2+^ was lower under acid condition. Accordingly, the high removal extent of Zn^2+^ by nZVI might be caused by adsorption and co-precipitation, which was proved by the effects of DO and pH as mentioned above. The formation of FeOOH on the surface of nZVI could be the main factor of Zn^2+^ removal because of its high adsorption affinity for aqueous solutes [32].

**Figure 7:**
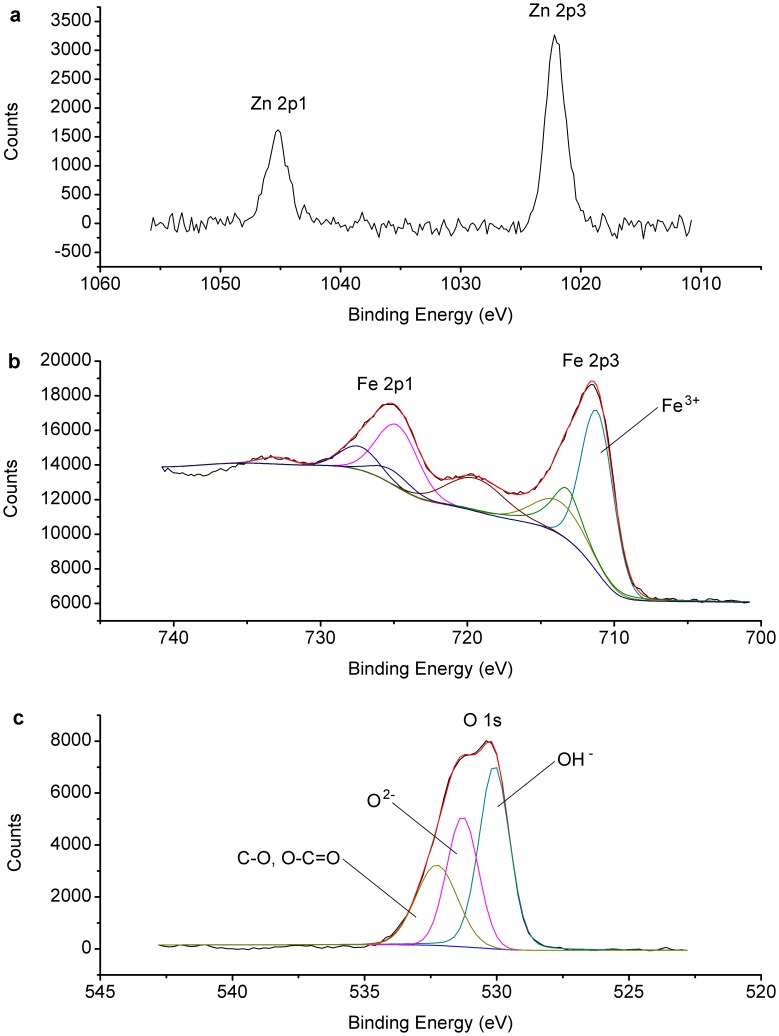
The XPS analysis of stock nZVI. The XPS narrow scan and curve fitting were analyzed: (a) the XPS narrow scan analysis of Zn 2p, (b) curve fitting analysis of Fe 2p, and (c) curve fitting analysis of O 1 s.

**Figure 8:**
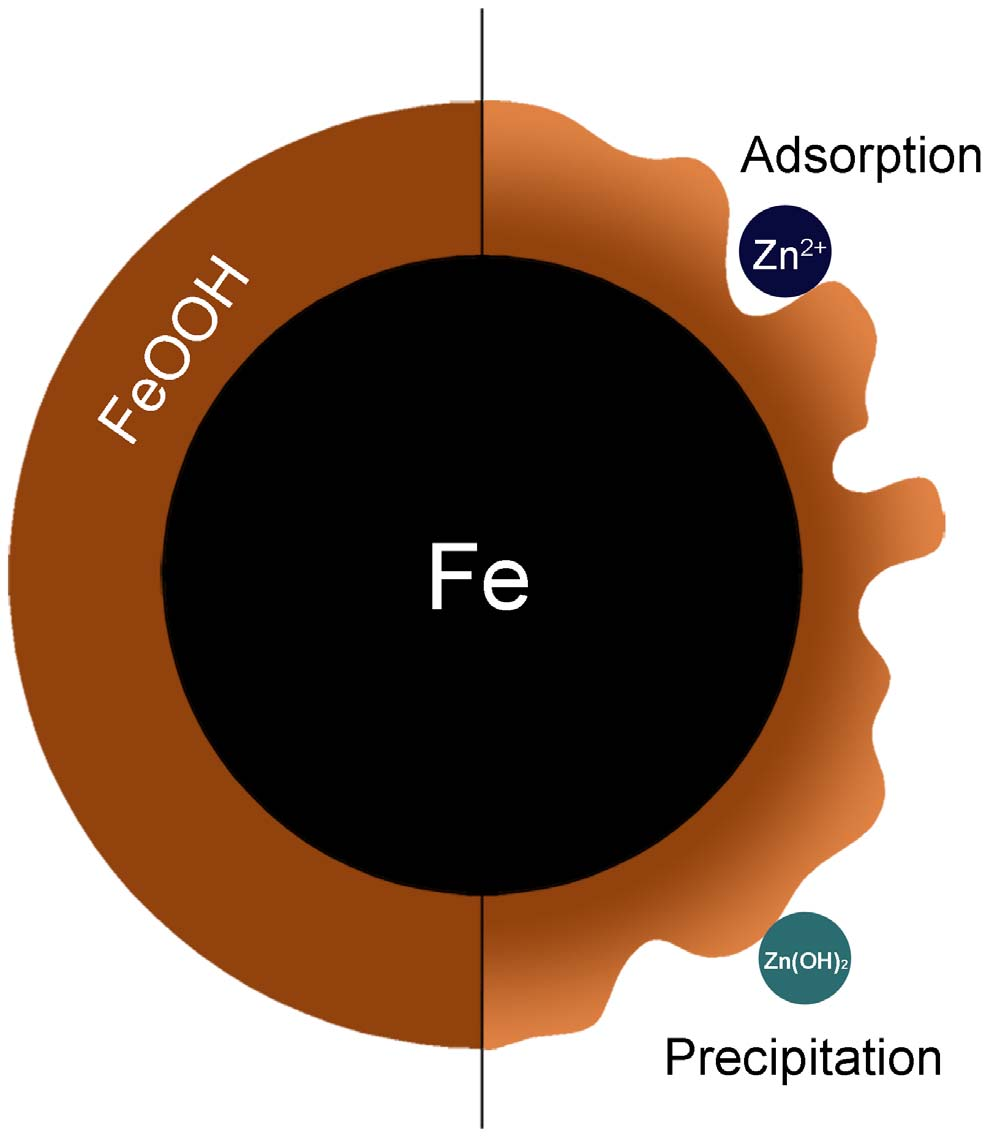
The structure of nZVI during reaction.

These phenomena could also be confirmed by XPS, as shown in Figure 7(a), (b) and (c). According the spectra of Figure 7(a), the binding energy of Zn 2p3 was 1022.2 eV, the difference between Zn 2p3 and 2p1 was 23.2 eV, which declared the Zn chemical shift [33]. And it was one way to identify the change of valence. The binding energy of LMM transition, the sharpest auger peak of zinc, was 498.2 eV. Compared with the auger parameters and the strongest photoelectron peak of zinc in handbook of X-ray [33], it could be determined that the zinc on the surface of nZVI was divalent [34]. According to the curve fitting analysis of Fe 2p and O 1 s, the peaks of Fe^3+^, OH^−^ and O^2−^ could be found [35], indicating the formation of FeOOH on nZVI surface. Accordingly, adsorption and co-precipitation are the most likely mechanism of Zn^2+^ removal by nZVI (Figure 8).

**Figure 9:**
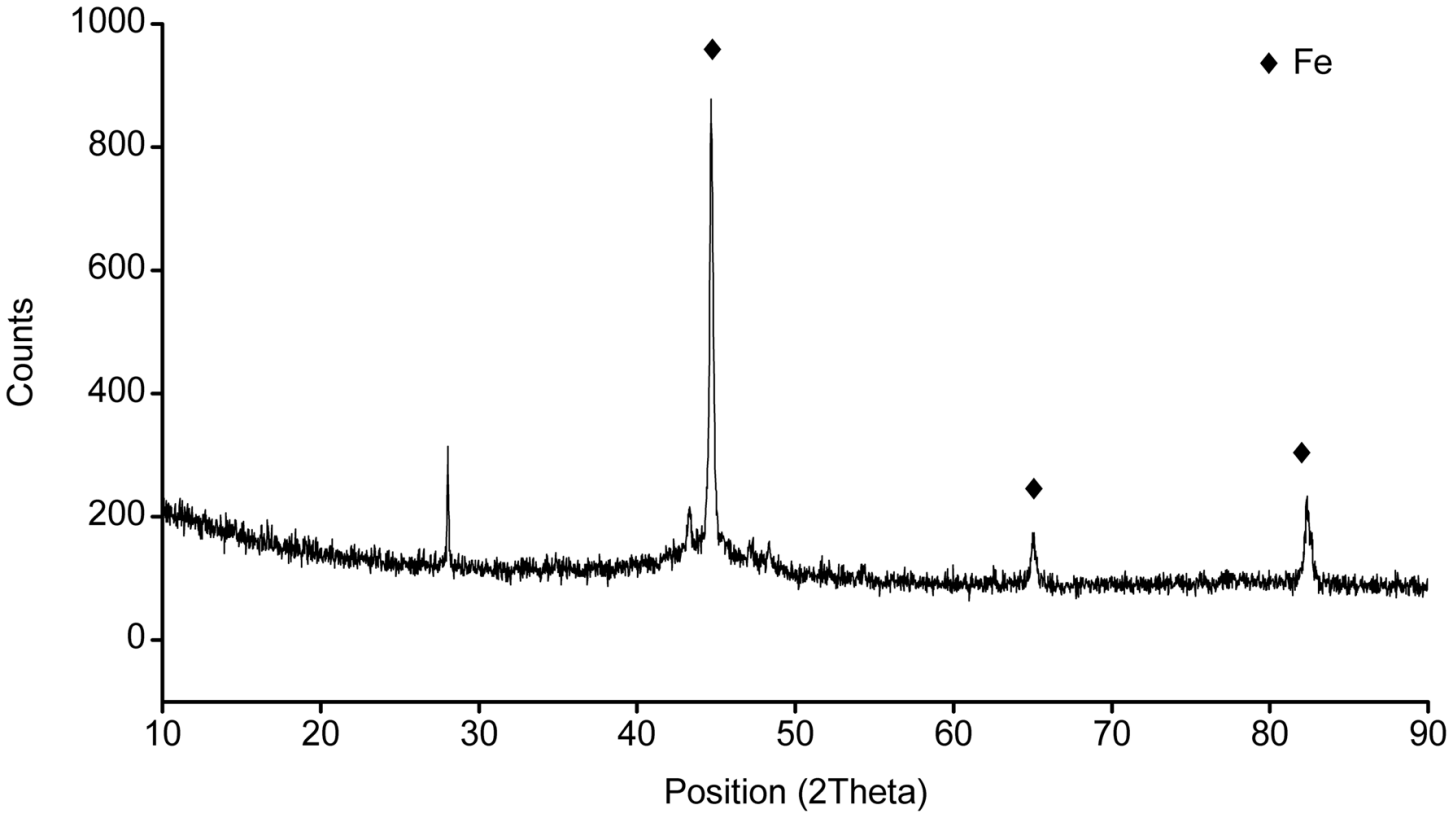
The XRD analysis of spent nZVI.

The nZVI is the core-shell structure: a single particle composed of a dense core surrounded by a thin amorphous shell exhibiting markedly less density than the interior core. As shown in Figure 2, the core-shell structure of nZVI could be found by TEM. The presence of Fe could be proved by XRD (Figure 9). The chemical composition of the passivated thin shell is believed to be a mixed Fe(II)/Fe(III) oxide phase [36], [37]. When nanoscale iron particles are exposed to water media, they will obtain hydroxide groups and consequently an apparent surface stoichiometry in proximity to FeOOH is formed [28]. The FeOOH-shell could enhance the adsorption. The H^+^ inhibited the formation of iron (oxy)hydroxide, resulting in the low removal extent of Zn^2+^ by nZVI.

### Effect of Cadmium

**Figure 10:**
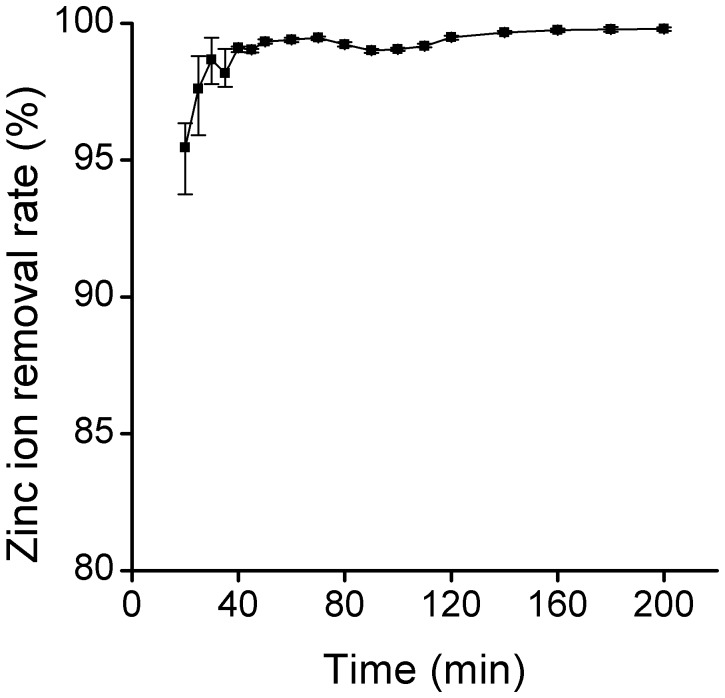
Simultaneous and individual removal of Zn^2+^ and Cd^2+^ by nZVI. Sample 1 contained 100/L Zn^2+^ solution. Sample 2 contained 100 mg/L mixture of Zn^2+^ Cd^2+^. Sample 3 contained 100 mg/L Cd^2+^ solution.

Simultaneous and individual removals of Zn^2+^ and Cd^2+^ by nZVI were shown in Figure 10. The removal extent of Zn^2+^ was much higher than that of Cd^2+^. Simultaneous removals of Zn^2+^ and Cd^2+^ by nZVI were both lower than individual removal. The Zn^2+^ removal extent was 0.8% lower in sample 2 than sample 1. And the Cd^2+^ removal efficiency by nZVI was 2.4% lower in mixed contaminants of sample 2 than sample 3. Hardiljeet’s research has proved that Cd^2+^ removal by nZVI was chemisorption [38]. So the selective behavior happened between Zn^2+^ and Cd^2+^ on nZVI. There could be the same adsorption sites for both Zn^2+^ and Cd^2+^. The removal efficiency and selectivity of nZVI particles for Zn^2+^ were higher than Cd^2+^.

### Removal of Zn^2+^ in Continuous Flow Reactor

**Figure 11:**
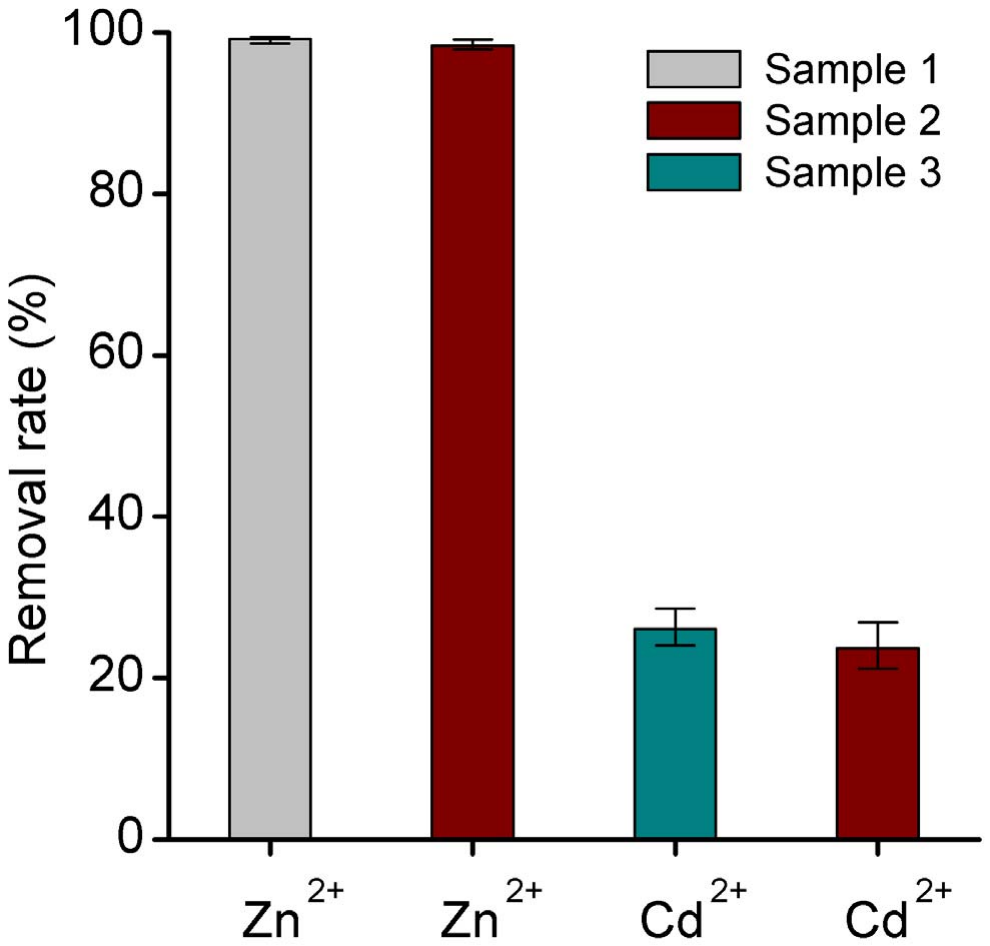
Removal of Zn^2+^ in continuous flow reactor.

The high removal efficiency of Zn^2+^ by nZVI was demonstrated by jar-test. The removal extent of 100 mg/L Zn^2+^could reach 85% by 1 g/L nZVI in 2 h, which should be adopted during reactor design and parameter control, such as influent flow, velocity, etc. The contact time of nZVI and Zn^2+^should be 2 h or more and the nZVI solids concentration should be no less than 1 g/L when the concentration of Zn^2+^ was 100 mg/L. The results of Zn^2+^ removal in continuous flow reactor were shown in Figure 11. The maximum removal efficiency was up to more than 95%, and the removal efficiency was steady after a rapid increase in the first 30 min. Furthermore, it should be found that this experiment may provide an applicable purification approach for water polluted by heavy metal for this technology allowed the enhanced reactivity and the favorable field deployment capabilities without secondary pollution of nZVI particles.

### Conclusions

This study demonstrated that the uptake of Zn^2+^ by nZVI was efficient. With the solids concentration of 1 g/L nZVI, more than 85% of Zn^2+^ could be removed within 2 h. The pH value and DO were the important factors of Zn^2+^ removal by nZVI. The DO enhanced the removal efficiency of Zn^2+^. Under the oxygen-contained condition, oxygen corrosion gave the nZVI surface a shell of iron (oxy)hydroxide, and the removal efficiency reached 80%, which could show high adsorption affinity. In contrast, the removal efficiency of Zn^2+^ was only 40% under oxygen-limiting condition. The removal efficiency of Zn^2+^ increased with the increasing of the pH. Acidic condition reduced the removal efficiency of Zn^2+^ by nZVI because the existing H^+^ inhibited the formation of iron (oxy)hydroxide. The higher removal efficiency was observed in the experiment with an initial pH value of 5. Adsorption and co-precipitation were the most likely mechanism of Zn^2+^ removal by nZVI. The FeOOH-shell could enhance the adsorption efficiency of nZVI. The removal extent of Zn^2+^ was much higher than that of Cd^2+^. The removal efficiency and selectivity of nZVI particles for Zn^2+^ were higher than Cd^2+^. Furthermore, a continuous flow reactor for engineering application of nZVI was designed and exhibited high removal efficiency for Zn^2+^. The maximum removal efficiency was up to more than 95%, and the removal efficiency was steady after a rapid increase in the first 30 min.
